# *Syzygium jambolanum *treatment improves survival in lethal sepsis induced in mice

**DOI:** 10.1186/1472-6882-8-57

**Published:** 2008-10-13

**Authors:** Márcia CG Maciel, Jardel C Farias, Michele J Maluf, Eliane A Gomes, Paulo VS Pereira, Walmir C Aragão-Filho, Josias B Frazão, Graciomar C Costa, Sanara M Sousa, Lucilene A Silva, Flávia MM Amaral, Momtchilo Russo, Rosane NM Guerra, Flávia RF Nascimento

**Affiliations:** 1 Laboratory of Immunophysiology. Federal University of Maranhão, São Luís, MA, Brazil; 2 Laboratory of Immunobiology. São Paulo University, São Paulo, SP, Brazil

## Abstract

**Background:**

The leaves and the fruits from *Syzygium jambolanum *DC.(Myrtaceae), a plant known in Brazil as sweet olive or 'jambolão', have been used by native people to treat infectious diseases, diabetes, and stomachache. Since the bactericidal activity of *S. jambolanum *has been confirmed *in vitro*, the aim of this work was to evaluate the effect of the prophylactic treatment with *S. jambolanum *on the *in vivo *polymicrobial infection induced by cecal ligation and puncture (CLP) in mice.

**Methods:**

C57Bl/6 mice were treated by the subcutaneous route with a hydroalcoholic extract from fresh leaves of *S. jambolanum *(HCE). After 6 h, a bacterial infection was induced in the peritoneum using the lethal CLP model. The mice were killed 12 h after the CLP induction to evaluate the cellular influx and local and systemic inflammatory mediators' production. Some animals were maintained alive to evaluate the survival rate.

**Results:**

The prophylactic HCE treatment increased the mice survival, the neutrophil migration to infectious site, the spreading ability and the hydrogen peroxide release, but decreased the serum TNF and nitrite. Despite the increased migration and activation of peritoneal cells the HCE treatment did not decrease the number of CFU. The HCE treatment induced a significant decrease on the bone marrow cells number but did not alter the cell number of the spleen and lymph node.

**Conclusion:**

We conclude that the treatment with *S. jambolanum *has a potent prophylactic anti-septic effect that is not associated to a direct microbicidal effect but it is associated to a recruitment of activated neutrophils to the infectious site and to a diminished systemic inflammatory response.

## Background

Many species have been used in the folk medicine to treat infectious diseases, and some of their anti-microbial activities have been proved. In fact, the anti-microbial activity has been recognized in different species of vegetal families, and this activity is usually due to the presence of secondary metabolites.

*Syzygium jambolanum *DC. (syn:*Syzygium cumini *Skeels, *Eugenia jambolana *Lam.) is commonly known in Brazil as 'jambolão', in India as Naval pazham and in English-speaking countries as jambolan, sweet olive or java plum. This species, from the myrtle family (Myrtaceae), has been used to treat illnesses caused by bacterial, fungal and viral pathogens [1], ulcers in genitourinary tract caused by *Candida albicans*, as well as cold, cough, fever and skin problems such as rashes and the mouth, throat and intestines [2]. In India, it has been used, in a mix with honey or milk, to treat diabetes and digestive diseases and the fresh fruits has been taken orally to treat stomachache [3].

The antimicrobial activity of *S. jambolanum *has been confirmed *in vitro *by some authors using bacteria strains [4-6] or *Leishmania *promastigotes [7]. However, there are no results about the effect of this species on the *in vivo *bacterial infection.

Based on this, the aim of this work was to evaluate the effect of the prophylactic treatment with a hydroalcoholic extract from fresh leaves of *S. jambolanum *in the lethal sepsis induced by cecal ligation and puncture in mice.

## Methods

### Mice

Male C57Bl/6 mice (10/group), eight to twelve-weeks-old, weighing 20–25 g have been maintained for many generations in the Animal Breeding Unit (*Biotério da Universidade Federal do Maranhão*, Sao Luis, MA, Brazil) under standard conditions. The animals were kept in well cross ventilated room at 26 ± 2°C, relative humidity 44–56%, light and dark cycles of 12 h. The animals had free access to sterilized food and acidified water. All procedures described were reviewed and approved by the Animal Ethics Committee in accordance with COBEA (Brazilian College of Animal Experimentation).

### Plant material

Leaves of *Syzygium jambolanum *DC. (Myrtaceae) were collected and identified at the Ático Seabra Herbarium of the Universidade Federal do Maranhão (São Luís, MA, Brazil) (voucher specimen N° 1087). The fresh leaves (200 g) were extracted with 1 L of ethanol (70%) and mixed every 8 h during 24 h. The same procedure was repeated four times. After this period the hydroalcoholic crude extract (HCE) was filtered using a cotton funnel and it was concentrated under low pressure. The yield obtained was 5.1% (w/w). Finally, the HCE was dried and the remainder was later lyophilized. For the experiments, the lyophilized dry residue was diluted in an isotonic phosphate buffered saline (PBS) at a concentration of 1 mg/mL. The animals were then weighed to adjust the dose of HCE to 5 and 50 mg/Kg (mg of dried plant material/Kg of body weight). These doses were chosen based in pilot experiments of neutrophil recruitment.

### Experimental design

Polymicrobial sepsis was induced using cecal ligation and puncture (CLP) according to previously described methods [8]. Briefly, under deep anesthesia, a laparotomy was performed and the cecum was mobilized and ligated below the cecal valve, punctured 8× with an 18-gauge needle to induce the lethal sepsis. The cecum was replaced into peritoneal cavity and the abdomen was closed in two layers. Saline (0.5 mL/10 g body weight) was given subcutaneously to CLP animals for fluid resuscitation.

The animals were initially divided into 4 groups that were treated by subcutaneous route 6 hours before the CLP induction. In group 1 (Control) the mice received a control vehicle (saline solution). In groups 2 and 3 the mice received the HCE treatment at the doses of 5 (HCE 5) or 50 mg/Kg (HCE 50), respectively. In group 4 (Sham), the cecum was not perforated and the mice were not treated.

To evaluate the lifespan the mortality of the animals was recorded every 12 h until the 5^th ^day. The mice which remained alive were followed by one month. In all the subsequent assays, the blood of anesthetized mice was collected 12 h after the CLP, and the animals immediately sacrificed.

### Blood Glucoses Concentration

The quantification of glucoses was made in peripheral blood using a digital glucosimeter (Advantage II – Roche) with specific material. The values obtained represent the concentration of glucoses (mg/dL).

### TNF bioassay

The serum TNF was measured by a modification of the standard L929 *in vitro *cytotoxicity assay using actinomycin D-treated target cells as previously described method [9].

### Determination of serum nitrite concentration

Serum NO levels were determined by the measurement of nitrite and nitrate after enzymatic reduction of nitrate with nitrate reductase, as previously described [10].

### Peritoneal cell harvesting

The peritoneal cell harvesting and the assays to evaluate the spreading, the hydrogen release and the nitric oxide production by peritoneal cells were performed according to previously described methods [11].

### Platelets, spleen, lymph node and bone marrow's cells counting

The platelets count and the lymphoid cells quantification were performed according to previously described methods [12].

### Colony forming units (CFU) determination

The mice were killed 12 hours after the CLP. The skin of the abdomen was cut open in the midline after thorough disinfection and without injury to the muscle and the peritoneal cavity was washed with 2 mL of sterile phosphate buffered solution (PBS). Aliquots of serial log dilutions of the peritoneal fluid obtained were plated on Mueller-Hinton agar dishes (Difco Laboratories, Detroit); colony-forming units were counted after overnight incubation at 37°C, and the results were expressed as log_10 _of the number of colony-forming units per peritoneal cavity.

### Histopathology

To evaluate the inflammatory infiltrate to the cecum walls from mice submitted to CLP, 3 animals from each group were killed 12 h after surgery; cecum fragments were removed, fixed in 10% phormol for 24 h, dehydrated in alcohol, and embedded in paraffin. Serial 5-mm sections were stained with hematoxylin-eosin for analysis of the inflammatory response.

### Statistical Analysis

Results are expressed as the mean ± standard error of mean (SEM) deviation from 10 animals per group. Statistical evaluation was done by ANOVA test followed by Neuman-Keuls. Mice lifespan was demonstrated using the Kaplan-Meier curve and the log-rank statistical test was applied to compare the curves. Differences were considered significant at P = 0.05 and are represented by an asterisk. All experiments were repeated for at least two times.

## Results

### Effect of prophylactic HCE treatment on survival in CLP-induced sepsis

**Figure 1:**
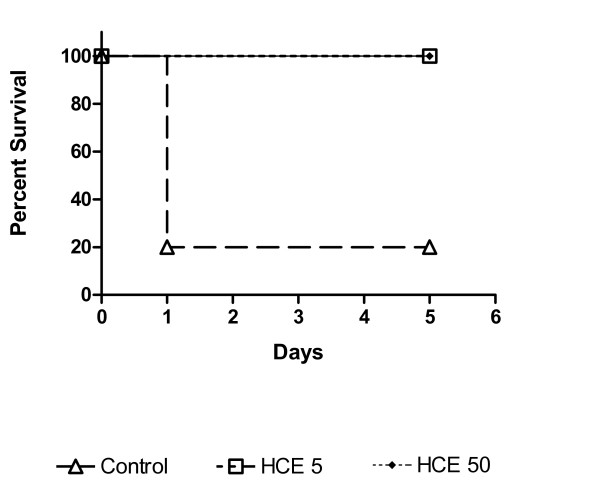
**Effect of *Syzygium jambolanum *HCE treatment on lethal sepsis induced by CLP.** The cecum was perforated 8× with an 18 G needle. The treatment with HCE at the doses of 5 (HCE 5) or 50 mg/Kg (HCE 50) was done 6 hours before the CLP. The mice survival was observed until the 5^th ^day. The results were expressed as mean ± S.E.M of 10 animal/group.

The CLP induced the death of 80% of the mice until the 24^th ^hour in the control group. However, the prophylactic HCE treatment improved the mice survival when compared to the control group (Figure 1). The survival was followed by one month and that mice which had survive until the 5^th ^day remains alive by all the period. Based on this result, in the rest of experiments, the mice were treated with HCE and killed 12 h after the CLP induction to investigate the mechanisms of protection.

### Effect of prophylactic HCE treatment on the cellular influx to peritoneal cavity induced by CLP

**Figure 2:**
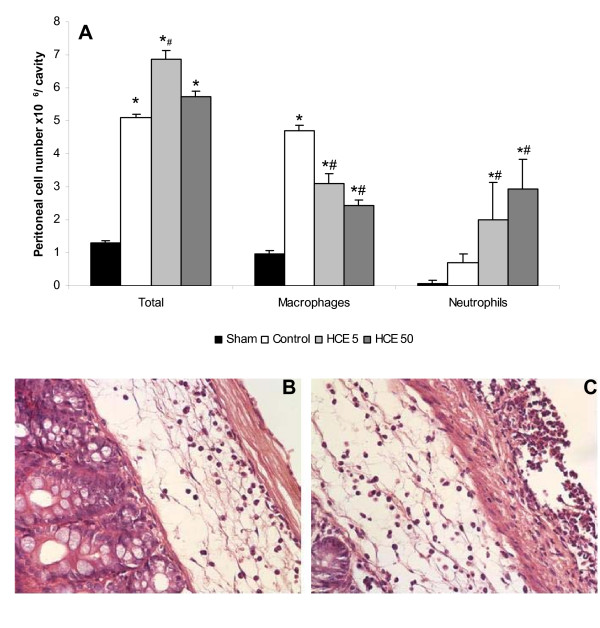
**Total and differential cell counting in the peritoneal cavity and inflammatory infiltration in the cecum wall.** The total and differential counting of peritoneal cells were performed 12 h after the CLP (A). The results were expressed as mean ± S.E.M (10 animals/group). The cecum of the control group (B) or of the HCE 5 group (C) was obtained to evaluate the histopathology at a 400× magnification. *p < 0.05 when compared to sham and # when compared to the control group.

The cell recruitment to the peritoneal cavity, constituted mainly by neutrophils, was enhanced in the HCE treated mice when compared to the control group (Figure 2A). The treatment with HCE also induced an intense infiltration of inflammatory cells to the cecum walls which was more evident than that observed in the control group (Figures 2B–C).

### Effect of prophylactic HCE treatment on the peritoneal cells activation induced by CLP

The HCE treatment significantly increased both the *ex vivo *spreading ability and the hydrogen peroxide release by peritoneal cells when compared to control group. Nevertheless, the HCE treatment did not change the *ex vivo *spontaneous NO production by peritoneal cells. On the other hand, the serum TNF and nitrate/nitrite were decreased in the HCE group when compared to the control group (Table 1).

**Table 1:** Effect of HCE prophylactic treatment on peritoneal cell activation and local and systemic inflammatory mediators in mice submitted to the CLP

	Control	HCE 5	HCE 50
Spreading index (%)	13.1 ± 2.0^a^	17.3 ± 3.3*	36.2 ± 3.1*
Spontaneous H_2_O_2 _release (μM)	10.4 ± 0.1	17.2 ± 0.2*	19.2 ± 0.2*
Nitrite in the culture supernatant (μM)	5.4 ± 0.7	6.3 ± 0.5	7.6 ± 0.5
Seric Nitrite (μM)	165.1 ± 2.5	155.3 ± 3.1*	127.7 ± 2.3*
Seric TNF (UL/mL)	4.0 ± 1.1	2.5 ± 0.5*	0.5 ± 0.2*

^a^The results were expressed as mean ± S.E.M (10 animals/group).

*p < 0.05 when compared to the control group.

The HCE treatment did not decrease the number of CFU in the peritoneal cavity fluid (Control: 5.6 ± 0.1, HCE 5: 5.5 ± 0.2, HCE 50: 5.5 ± 0.1 Log_10_)

### Effect of prophylactic HCE treatment on lymphoid organs cellularity and on platelets counting in mice submitted to CLP

The HCE treatment induced a significant decrease on the bone marrow cells number and in the platelets count in peripheral blood when compared to the control group. However, it did not alter the cell number in both the spleen and the lymph node. (Table 2).

**Table 2:** Effect of HCE prophylactic treatment on lymphoid organs cellularity and platelets counting in mice submitted to the CLP

	Control	HCE 5	HCE 50
Bone marrow (x10^6^/mL)	9.5 ± 0.1^a^	8.7 ± 0.1	5.6 ± 0.1*
Spleen (x10^6^/mL)	56.7 ± 0.2	55.4 ± 1.7	55.8 ± 1.1
Lymph node (x10^6^/mL)	12.3 ± 0.6	9.7 ± 0.2*	10.3 ± 0.3*
Platelets (x10^6^/mL)	56.8 ± 1.3	51.8 ± 2.8	35.2 ± 1.3*

^a^The results were expressed as mean ± S.E.M of cell number (10 animals/group).

*p < 0.05 when compared to the control group

### Effect of prophylactic HCE treatment on glucose blood levels from mice submitted to CLP

The CLP induced, *per se*, a decrease in the blood glucose levels after 12 hours, what was observed in the control and in the HCE-treated groups when compared to the sham group (Sham: 94.3 ± 1.5, Control: 46.1 ± 6.7 mg/dL). The HCE prophylactic treatment did not alter the glucose levels when compared to the control group (Control: 46.1 ± 6.7, HCE 5: 44.1 ± 2.9, HCE 50: 36.3 ± 5.1).

## Discussion

Some *in vitro *evidences demonstrated that *S. jambolanum *has a microbicidal activity [2-7]. To evaluate the *in vivo *anti-microbial effect of this species we used the model of CLP which resembles the clinical situation of bowel perforation and mixed bacterial infection of intestinal origin which seems to be the most realistic model of sepsis.

The present study demonstrated that the prophylactic treatment with HCE from the leaves of *S. jambolanum *effectively reduces the mortality of CLP-induced lethal sepsis in mice. However, this effect was not related to a direct anti-microbial effect of the HCE since the CFU was not decreased. In fact, the HCE was used by subcutaneous route, so, it had no direct contact with the bacteria in the peritoneum, what can justify the absence of a microbicidal effect in the first hours post CLP. It is reasonable to suppose that 12 hours post CLP the bacteria were already not controlled in the initial focus. However, the HCE can improve the survival because it is able to induce a neutrophil recruitment to the initial focus of infection and also to reduce the systemic inflammation. The latter results are in accordance with previous relates which shown that this extract has an anti-inflammatory effect [13].

Neutrophil recruitment to the infection site is, indubitably, an essential step to the control of bacterial infections because these cells are able to phagocyte and produce free radicals that ultimately kill the microorganisms [8,14-16]. We showed here that the cells recruited to the peritoneal cavity from HCE-treated mice showed a significant increase in both the spreading ability and the hydrogen peroxide release, which means an enhanced potential to kill microorganisms. A similar result was obtained recently using *Acanthus montanus *in the treatment of furuncles. It was shown that this species has antimicrobial properties which are directly related to its ability to recruit leukocytes to the infection site [17].

Despite the HCE treatment has induced an increase of hydrogen peroxide; the production of nitric oxide (NO) by recruited cells was not altered. On the other hand, there was a significant decrease of NO and TNF serum levels in HCE group when compared with control group. A similar result was obtained using Liu-Shen-Wan, a Chinese plant used as a traditional medicine to treat infectious diseases. The authors also found an increased survival of mice what was associated with an increase in the neutrophil recruitment and an inhibition of systemic inflammation [18].

The decrease of NO can be one of the mechanism related to the improved survival in the HCE-treated group, since the exacerbated production of NO could result in decrease of neutrophil recruitment [14], and also could induce a vase relaxation which results in the hypotension that exacerbate the sepsis.

One of the consequences in the sepsis is the hypoglycemia [19-21]. In fact, we found that CLP induced *per se *a decrease in the blood glucose levels which was not aggravated by *S. jambolanum *treatment.

## Conclusion

In conclusion, the results obtained here show that the HCE of the leaves from *S. jambolanum *has a prophylactic effect in the sepsis induced by CLP. This effect is related to an increased migration of neutrophil to the primary infection focus and also to the activation of those cells. These effects could justify the popular use of this plant in the treatment of infectious diseases. Further studies are in progress in order to characterize the bioactive compounds involved in the biological action of *S. jambolanum*.

## Abbreviations

CLP: cecal ligation and puncture; HCE: hydroalcoholic crude extract; H_2_O_2_: hydrogen peroxide; NO: nitric oxide; PBS: phosphate buffered saline; TNF: tumor necrosis factor; sc.: subcutaneous.

## Competing interests

The authors declare that they have no competing interests.

## Authors' contributions

MCGM designed the experiments, carried out the CLP, performed the statistical analysis, and drafted the manuscript. JCF performed the CLP, and quantified the platelets. EAG carried out the NO and TNF assays. PVSP, WCAF, and JBF carried out the immunoassays. LAS carried out the histopathological analysis and performed the photos. MJM, GCC, SMS, FMMA collected the leaves and prepared the extract and suspensions. MR and RNMG participated in the design and discussions, and corrected the manuscript. FRFN conceived of the study, and participated in its design and coordination. All authors read and approved the final manuscript.

## Pre-publication history

The pre-publication history for this paper can be accessed here:


